# Two CD9 tetraspanin family members of Japanese flounder (*Paralichthys olivaceus*): characterization and comparative analysis of the anti-infectious immune function

**DOI:** 10.1186/s13567-021-00903-3

**Published:** 2021-02-17

**Authors:** Jiaojiao He, Hanjie Gu, Wenqi Wang, Yonghua Hu

**Affiliations:** 1 Marine Science and Engineering College, Qingdao Agricultural University, Qingdao, 266109 China; 2 Institute of Tropical Bioscience and Biotechnology, Hainan Academy of Tropical Agricultural Resource, CATAS, Haikou, 571101 China; 3 Laboratory for Marine Biology and Biotechnology, Pilot National Laboratory for Marine Science and Technology (Qingdao), Qingdao, 266071 China; 4 Hainan Provincial Key Laboratory for Functional Components Research and Utilization of Marine Bioresources, Haikou, 571101 China

**Keywords:** CD9, *Paralichthys olivaceus*, Anti-infectious immunity, Pathogen

## Abstract

CD9 is a glycoprotein of the transmembrane 4 superfamily that is involved in various cellular processes. Studies related to the immune functions and activities of CD9 in teleost fish are limited. In this study, we characterized two CD9 homologs, PoCD9.1 and PoCD9.3, from Japanese flounder (*Paralichthys olivaceus*). Sequence analysis showed that PoCD9.1 and PoCD9.3 possess characteristic transmembrane 4 superfamily (TM4SF) structures. PoCD9.1 shares 70.61% sequence identity with PoCD9.3. The expression of *PoCD9.1* and *PoCD9.3* in the three main immune tissues was significantly induced in a time-dependent manner by extracellular and intracellular pathogen infection, which indicates that the two CD9 homologs play an important role in the response to pathogenic infection. Following infection with the extracellular pathogen *Vibrio anguillarum*, the expression profiles of both *PoCD9.1* and *PoCD9.3* were similar. After infection with the intracellular pathogen *Edwardsiella piscicida*, the expression levels of *PoCD9.1* and *PoCD9.3* were different at different stages of infection, especially in the spleen. The spleen was the most important tissue for the PoCD9.1 and PoCD9.3 responses to pathogen infection among the three examined immune tissues. Knockdown of *PoCD9.1* and *PoCD9.3* attenuated the ability of host cells to eliminate pathogenic bacteria, and *PoCD9.1* knockdown was more lethal than *PoCD9.3* knockdown for host cells with *E. piscicida* infection. Overexpression of *PoCD9.1* and *PoCD9.3* promoted host or host cell defence against *E. piscicida* infection. These findings suggest that PoCD9.1 and PoCD9.3 serve as immune-related factors, play an important role in the immune defence system of Japanese flounder, and display different functions in response to different pathogens at different stages of infection.

## Introduction

Aquaculture is one of the most rapidly expanding farming systems worldwide. This rapid expansion has led to an increase in several pathogens that infect a wide variety of fish species. *Edwardsiella piscicida* is one of the most important fish pathogens. *E. piscicida* (formerly known as *E. tarda*) [1, 2], belonging to the Enterobacteriaceae family, is the aetiological agent of edwardsiellosis in fish and a major problem in global aquaculture [3, 4]. *E. piscicida* has a broad host range and has been isolated from more than 20 fish species, such as ayu (*Plecoglossus altivelis*), barramundi (*Lates calcarifer*), blackspot seabream (*Pagellus bogaraveo*), catfish (*Ictalurus punctatus*), European eel (*Anguilla anguilla*), grouper *(Epinephelus aeneus*), Japanese eel (*A. japonica*), Japanese flounder (*Paralichthys olivaceus*), koi (*Cyprinus carpio*), rainbow trout (*Oncorhynchus mykiss*), red seabream (*Pagrus major*), tilapia (*Tilapia nilotica*), turbot (*Scophthalmus maximus*), and whitefish (*Coregonus lavaretus*) [4, 5]. The occurrence of disease is actually an incessant race between the pathogen and the host. The innate immune system is an extremely important defence mechanism of fish against pathogenic infection. For example, turbot limits *E. piscicida* colonization in vivo using interleukin-1β [6]. Flounder suppresses *E. piscicida* infection by regulating autophagy [7]. Pathogens also develop a variety of strategies to cope with host immune defence. For example, with the help of thioredoxin-like proteins, *E. piscicida* inhibits ASK1-MAPK signalling cascades to promote pathogenesis during infection [8]. To clearly clarify the host defence mechanism and control disease occurrence, researchers should identify and study more immune-related factors.

CD9 is a member of the tetraspanins or transmembrane 4 superfamily (TM4SF). Tetraspanins are structurally characterized by four transmembrane domains, which delimit a small extracellular loop (SEL), a large extracellular loop (LEL) with a CCG motif, and two short intracellular termini [9]. Compared with other members of the tetraspanin family, CD9 is peculiar since it contains only one N-glycosylation site that is located in its SEL domain [10]. CD9 was shown to be involved in many physiological and pathological processes. Reports have demonstrated that CD9 is implicated in the motility, adhesion, and activation of cells and hence plays a pivotal role in tumour growth, sperm-egg fusion, virus susceptibility, and tumour metastasis [11–17].

Characterization of CD9, initially identified as a lymphohaematopoietic marker [18], revealed its expression on various haematopoietic and nonhaematopoietic cells and showed it had specific functions on leukocytes, especially B cells, T cells, macrophages, dendritic cells, and eosinophilic cells [19–23], indicating that CD9 plays an active role in mammalian immune systems. With the deepening of research, the association between CD9 and other immune molecules, such as CD5, CD28, and MHC class II, has been documented [22–25], which prompted us to explore the functional mechanism of CD9 in the immune process. Specifically, CD9 can maintain the stabilization of immune synapses and the subsequent activation of T lymphocytes by regulating the clustering and adhesive activity of integrins of T cells [26, 27]. However, CD9 regulates the expression of molecules that can activate leukocyte cell adhesion and the avidity of its interaction with CD6 of antigen-presenting cells [28, 29]. Notably, CD9 is the only tetraspanin associated directly with ADAM17, a kind of disintegrin and metalloproteinase, on leukocytes and endothelial cells [30].

Compared to those in mammals, relatively few molecules involved in teleost immune responses have been identified and characterized. CD9 has been identified in a few species, including rainbow trout (*O. mykiss*) and Atlantic salmon (*Salmo salar*) [31, 32]. According to a previous study, the expression of CD9 was induced by pathogenic infection, which indicates that CD9 participates in the teleost immune response [32]. Japanese flounder is an economically important species of marine flatfish that is farmed worldwide, especially in Asian countries such as Japan, Korea, and China. Currently, the major economic losses in flounder culture caused by bacteria and viruses have become a serious problem that restricts the culture industry. Moreover, studies of the responses of CD9 isoforms to pathogenic infection are limited. Therefore, work committed to explaining the function and activity of flounder CD9 is important. According to the National Center for Biotechnology Information (NCBI) database, there are three isoforms of CD9 in *P. olivaceus*: CD9 antigen-like isoform X1 (XP_019934457.1), CD9 antigen-like isoform X2 (XP_019934458.1), and CD9 antigen-like X3 (XP_019964847.1). The sequence identity between isoforms X1 and X2 is 96.48%, and only eight amino acids are different at the N terminus. However, CD9 antigen-like X3 shares only 70.61% sequence identity with X1. In this study, CD9 antigen-like isoform X1 and CD9 antigen-like X3 (named PoCD9.1 and PoCD9.3 for convenience, respectively) from Japanese flounder were selected to analyse the immune functions. Our results showed that the expression of *PoCD9.1* and *PoCD9.3* was induced by different pathogens, and the two CD9s served as immune-related factors to promote host resistance against pathogenic infection. These findings will help further elucidate the biological functions of teleost CD9 in innate immunity.

## Materials and methods

### Fish

Clinically healthy Japanese flounders (average weight 16.5 ± 2.5 g) were purchased from a commercial fish farm in Shandong Province, China, and maintained at 22–24 °C in aerated seawater. Before the experiments, the fish were acclimatized in the laboratory for two weeks and verified to be free of pathogens in the liver, head kidney, and spleen, as reported previously [33]. For tissue collection, the fish were euthanized with tricaine methanesulfonate (Sigma-Aldrich Corporation, St. Louis, MO, USA), as reported previously [34].

### Bacterial and viral strains and host cells

The fish pathogen *E. piscicida* (formerly known as *E. tarda*) has been reported previously [35]. *Vibrio anguillarum* and fish infectious spleen and kidney necrosis virus (ISKNV) were kindly provided by Dr. Min Zhang of Qingdao Agricultural University, and viral proliferation was reported previously [36]. *Escherichia coli* was purchased from Transgene (Beijing, China). Bacterial strains were cultured in Luria–Bertani broth (LB) medium at 37 °C (for *E. coli*) or at 28 °C (all other microbes).

The FG cell line FG-9307 was derived from the gills of Japanese flounder and maintained according to the method described by Tong [37]. Briefly, FG cells were cultured in Leibovitz’s L-15 with L-glutamine (L-15; Solarbio Science & Technology Co., Ltd., Beijing, China) supplemented with 10% bovine calf serum (BCS; HyClone, Utah, USA), 100 IU mL penicillin and 100 mg/mL streptomycin in plastic culture flasks (Corning, New York, USA) at 23 °C.

### Sequence analysis

The sequences of PoCD9.1 (Gene ID:109,624,332) and PoCD9.3 (Gene ID:109,644,010) were obtained by PCR from flounder head kidney cDNA based on the sequences in the NCBI with the primers PoCD9.1F/PoCD9.1R and PoCD9.3F/PoCD9.3R (Table 1), which were designed by Primer Premier 5. The sequence was analysed using the BLAST program at the NCBI. The domain was searched using the simple modular architecture research tool. The calculated molecular mass and theoretical isoelectric point were predicted by the Compute pI/Mw tool. The cartoon diagram was manufactured by Protter. The phylogenetic tree was constructed based on the amino sequence alignment by the neighbour-joining (NJ) method with MEGA 7.0 software, and bootstrap trials were replicated 1000 times.

**Table 1:** Primers used in this study.

Primer name	Sequence (5′–3′)
PoCD9.1F	GTCGACATGGCCGCGCTGTCGG (SalI)
PoCD9.1R	CTCGAGCACCACCTCCCGAGATTTCC (XhoI)
PoCD9.3F	GTCGACATGGCGGCTCTGACCAGCT (SalI)
PoCD9.3R	CTCGAGGACAAAGTCTCTGGACCTCTTG (XhoI)
PoCD9.1RTF	TATGCTGGGACTGTTCTTTGG
PoCD9.1RTR	GCAGCAGTTCAGCCCTGTTT
PoCD9.3RTF	TGGTCGTCGGCTTCCTCG
PoCD9.3RTR	CCTTCAGGGCTTCTTGTTTGG
siPoCD9.1-F1	GATCACTAATACGACTCACTATAGGGCCTGGTGTTCTGGCTTGCATT
siPoCD9.1-R1	AATGCAAGCCAGAACACCAGGCCCTATAGTGAGTCGTATTAGTGATC
siPoCD9.1-F2	AACCTGGTGTTCTGGCTTGCACCCTATAGTGAGTCGTATTAGTGATC
siPoCD9.1-R2	GATCACTAATACGACTCACTATAGGGTGCAAGCCAGAACACCAGGTT
siPoCD9.1-CF1	GATCACTAATACGACTCACTATAGGGATCGCGTCGTCGTCGTGTTTT
siPoCD9.1-CR1	AAAACACGACGACGACGCGATCCCTATAGTGAGTCGTATTAGTGATC
siPoCD9.1-CF2	AAATCGCGTCGTCGTCGTGTTCCCTATAGTGAGTCGTATTAGTGATC
siPoCD9.1-CR2	GATCACTAATACGACTCACTATAGGGAACACGACGACGACGCGATTT
siPoCD9.3-F1	GATCACTAATACGACTCACTATAGGGGGAGACGCTGCGTCTCATCTT
siPoCD9.3-R1	AAGATGAGACGCAGCGTCTCCCCCTATAGTGAGTCGTATTAGTGATC
siPoCD9.3-F2	AAGGAGACGCTGCGTCTCATCCCCTATAGTGAGTCGTATTAGTGATC
siPoCD9.3-R2	GATCACTAATACGACTCACTATAGGGGATGAGACGCAGCGTCTCCTT
siPoCD9.3-CF1	GATCACTAATACGACTCACTATAGGGATCGATCGATCGTCGTCTCTT
siPoCD9.3-CR1	AAGAGACGACGATCGATCGATCCCTATAGTGAGTCGTATTAGTGATC
siPoCD9.3-CF2	AAATCGATCGATCGTCGTCTCCCCTATAGTGAGTCGTATTAGTGATC
siPoCD9.3-CR2	GATCACTAATACGACTCACTATAGGGGAGACGACGATCGATCGATTT
Poβ-actinRTF	GGACATCCGTAAGGACCTGT
Poβ-actinRTR	GCCTCCGATCCATACAGAGT
EFα-1RTF	CTACAAGTGCGGAGGAATCG
EFα-1RTF	GTCCAGGAGCGTCAATGATG

### *Quantitative real-time reverse transcription-PCR *(*RT-qPCR*)* analysis of PoCD9.1 and PoCD9.3 expression under normal conditions*

RT-qPCR analysis of *PoCD9.1* and *PoCD9.3* expression under normal conditions was performed as follows. Total RNA from the spleen, liver, head kidney, blood, intestine, muscle, gill, and brain of five fish was extracted using an EZNA Total RNA Kit (Omega Bio-tek, Doraville, GA, USA). Total RNA was treated with DNase I to remove residual DNA. One microgram of total RNA was used for cDNA synthesis with a RevertAid First Strand cDNA Synthesis Kit (Thermo Scientific, USA). RT-qPCR was performed using a Roche LightCycler 96 system (Switzerland) with a SYBR ExScript RT-qPCR Kit (TaKaRa Biotechnology Co., Ltd., Dalian, China) [38]. PCR was performed in a 20 μL volume containing 10 μL of SYBR® Premix Ex Taq™, 0.2 μM of each specific primer pair PoCD9.1RTF/R and PoCD9.3RTF/R, and 2 μL of diluted cDNA (100-fold dilution). The PCR conditions were 95 °C for 30 s, followed by 40 cycles of 95 °C for 15 s, 60 °C for 15 s, and 72 °C for 20 s. Melting curve analysis of the amplification products was performed at the end of each PCR to confirm that only one product was amplified. The expression levels of *PoCD9.1* and *PoCD9.3* were analysed using the comparative threshold cycle method (2^−ΔΔCT^) with beta-actin as an internal reference [39, 40]. The experiment was performed in triplicate.

### PoCD9.1 and PoCD9.3 expression upon bacterial and viral infection

RT-qPCR analysis of *PoCD9.1* and *PoCD9.3* expression during pathogen infections was performed as reported previously [41]. *V. anguillarum* and *E. piscicida* were cultured in LB broth at 28 °C to an optical density of 0.8 at 600 nm. Then, the cells were washed with phosphate-buffered saline (PBS) and resuspended in PBS to a concentration of 5 × 10^6^ CFU (colony forming units)/mL. ISKNV was resuspended in PBS to a concentration of 1 × 10^6^ copies/mL. The fish were divided randomly into four groups (16 fish per group) and injected intraperitoneally with 50 μL of *V. anguillarum*, *E. piscicida*, ISKNV, or PBS. After infection, the head kidney, spleen, and liver from three or four fish were taken aseptically at 6, 12, 24, 48, and 72 hours post-infection (hpi) for bacterial infection and at 1, 3, 5, and 7 days post-infection (dpi) for viral infection. *PoCD9.1* and *PoCD9.3* expression was determined by RT-qPCR, as described in the “Quantitative real-time reverse transcription-PCR (RT-qPCR) analysis of PoCD9.1 and PoCD9.3 expression under normal conditions” section. To determine the stability of beta-actin as an internal reference, we detected the expression of beta-actin with EF1 alpha as an internal reference during bacterial and viral infections. The RT-qPCR results showed that beta-actin expression in the three examined tissues upon infection with different pathogens was not significantly changed (Additional file 1), indicating that beta-actin is stable under the current experimental conditions. The experiment was performed in triplicate.

### PoCD9.1 and PoCD9.3 knockdown and its effects on bacterial infection

*PoCD9.1* and *PoCD9.3* were knocked down by injecting the synthesized siRNA as reported previously [42]. The siRNAs PoCD9.1-Ri and PoCD9.3-Ri were synthesized with an in vitro Transcription T7 Kit (for siRNA Synthesis) (TaKaRa Biotechnology Co.). For *PoCD9.1*, siPoCD9.1-F1/R1 and siPoCD9.1-F2/R2 (Table 1), containing the target sequence plus the T7 RNA polymerase promoter sequence and 6 extra nucleotides upstream of the minimal promoter sequence, were designed to obtain two DNA oligonucleotides after incubation at 95 °C for 2 min. Then, the templates were allowed to cool down at 25 °C for 45 min and maintained for 10 min. Next, the two DNA oligonucleotides were used for transcription in vitro at 42 °C for 2 h according to the manufacturer’s instructions. The DNA template was removed from the separate short RNA strands by digestion with DNase I. Finally, the synthesized siRNA was purified according to the manufacturer’s instructions. The control siRNA (PoCD9.1-RiC) was synthesized with two pairs of primers, siPoCD9.1-CF1/R1 and siPoCD9.1-CF2/R2 (Table 1), as described above. A similar synthetic process was performed for *PoCD9.3*, and PoCD9.3-Ri as well as PoCD9.3-RiC were obtained.

Transfection was performed as reported previously [43]. Briefly, FGs were distributed into two 96-well culture plates (1 × 10^5^ cells/well) in L-15 medium with 10% bovine calf serum, 100 IU mL penicillin and 100 μg/mL streptomycin (80–90% wall adherence rate). Then, the medium was replaced with L-15 without FBS and antibiotics. Transfection of the cells with PoCD9.1-Ri, PoCD9.1-RiC, PoCD9.3-Ri, PoCD9.3-RiC, or PBS (control) was performed with Lipofectamine 2000 (Invitrogen, Carlsbad, CA, USA) according to the manufacturer’s instructions. Briefly, 100 μL of mixture containing 0.5 μL of Lipofectamine 2000 and 0.4 μg siRNA or PBS was added to each well. After transfection for 24 h, one plate was used to assess the expression of *PoCD9.1* and *PoCD9.3*, and the cells were collected for RNA isolation. Another plate was replaced with new medium containing 1 × 10^6^ *E. piscicida*. The plate was incubated at 23 °C for 6 h and then washed three times with PBS. The cells were lysed by adding 100 μL of 1% Triton X-100 to each well. The lysate was diluted serially and plated on LB agar plates. The plates were incubated at 28 °C for 24 h, and the colonies that emerged on the plates were counted. The experiment was performed three times.

### Plasmid construction

The pCN3 [44] ampicillin resistance plasmid was derived from pCI-neo (Promega, USA), a mammalian expression vector, and contains the human cytomegalovirus immediate-early enhancer/promoter, which promotes constitutive expression of cloned DNA inserts in eukaryotic cells. The coding sequences of PoCD9.1 and PoCD9.3 amplified with the primers PoCD9.1F/R and PoCD9.3F/R (Table 1) were inserted into pEASY-Simple-T (TransGen Biotech, Beijing, China), and the genes were retrieved from the recombinant plasmids by digestion with XhoI and inserted into pCN3 at XhoI. The positive clones of the recombinant plasmid were screened by PCR on ampicillin-resistant plates and confirmed by sequencing. The recombinant plasmids were named pCNPoCD9.1 and pCNPoCD9.3, expressing PoCD9.1 and PoCD9.3, respectively. An Endo-Free Plasmid Kit (Omega Bio-tek, Doraville, USA) was used to prepare endotoxin-free plasmid DNA.

### Effects of PoCD9.1 and PoCD9.3 overexpression on bacterial infection

For analysis of the effect of *PoCD9.1* overexpression, pCNPoCD9.1 and pCN3 were transfected into FG cells as described above. After transfection for 24 h, the cells were infected with *E. piscicida* for 6 h. The bacterial number was determined as described above. For *PoCD9.3*, the effect of *PoCD9.3* overexpression was examined in vivo, and thirty fish were divided into two groups: pCNPoCD9.3 and pCN3 diluted in PBS (200 μg/mL). The fish were injected intramuscularly (i.m.) with 50 μL of pCNPoCD9.3 and pCN3. At 5 days after plasmid administration, spleen and muscle tissues of 5 fish from each group were collected for RNA and DNA extraction. The remaining fish were infected with *E. piscicida* as described above. Spleens from five fish were taken under aseptic conditions at 24 and 48 hpi. The bacterial number in the tissues was determined by plate count [45]. Briefly, the tissues were homogenized in PBS, and the homogenates were diluted serially. Then, the dilutions were plated on LB agar plates. The plates were incubated at 28 °C for 24 h, and the colonies that appeared on the plates were enumerated. The experiment was performed three times.

### Statistical analysis

All statistical analyses were performed with SPSS 18.0 software (SPSS, Inc., Chicago, IL, USA). Data were analysed with analysis of variance (ANOVA). Data are expressed as the mean ± standard error of the mean (SEM). Error bars indicate the SEM (*n* = 3, biologically independent samples). Statistical significance was defined as *P* < 0.05.

## Results

### Sequence analyses and structural characteristics of PoCD9.1 and PoCD9.3

**Figure 1:**
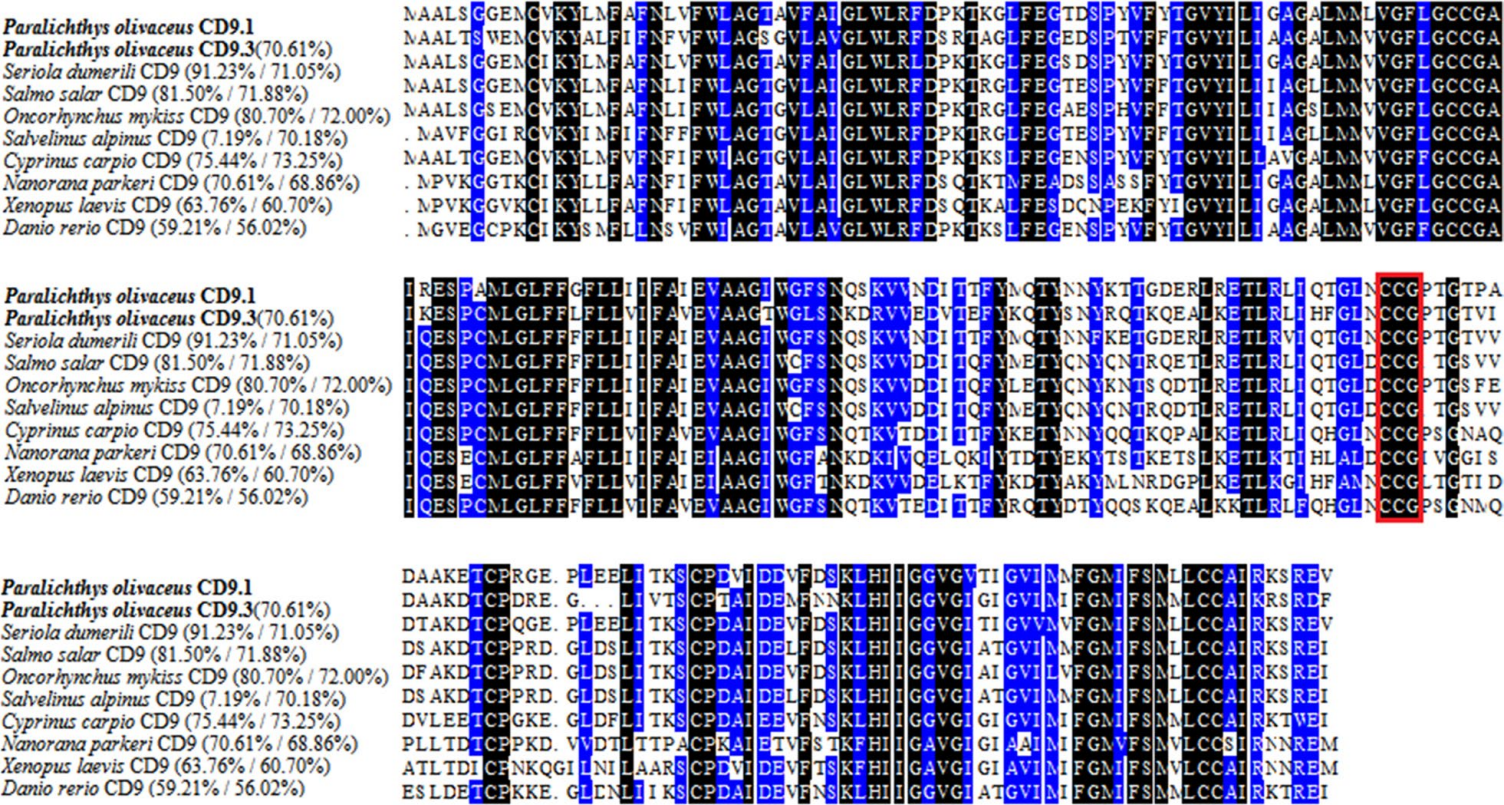
**Multiple sequence alignment of PoCD9.1 and PoCD9.3 homologues.** The percentage number in the bracket following each species name represents the overall sequence identity between PoCD9.1 or PoCD9.3 with other specified species. The consensus residues are in black, and the residues that are ≥ 75% identical among the aligned sequences are in dark blue. The CCG motif and conserved cysteine residues are boxed. The GenBank accession numbers of the aligned sequences are as follows: *Paralichthys olivaceus* CD9.1 (XP_019934457.1), *P. olivaceus* CD9.3 (XP_019964847.1), *Seriola dumerili* (XP_022610189.1), *Salmo salar* (XP_014003925.1), *Oncorhynchus mykiss* (XP_021428108.1), *Salvelinus alpinus* (XP_023827461.1), *Cyprinus carpio* (KTF92709.1), *Nanorana parkeri* (XP_018418195.1), *Xenopus laevis* (NP_001085461.1), and *Danio rerio* (NP_997784.1).

**Figure 2:**
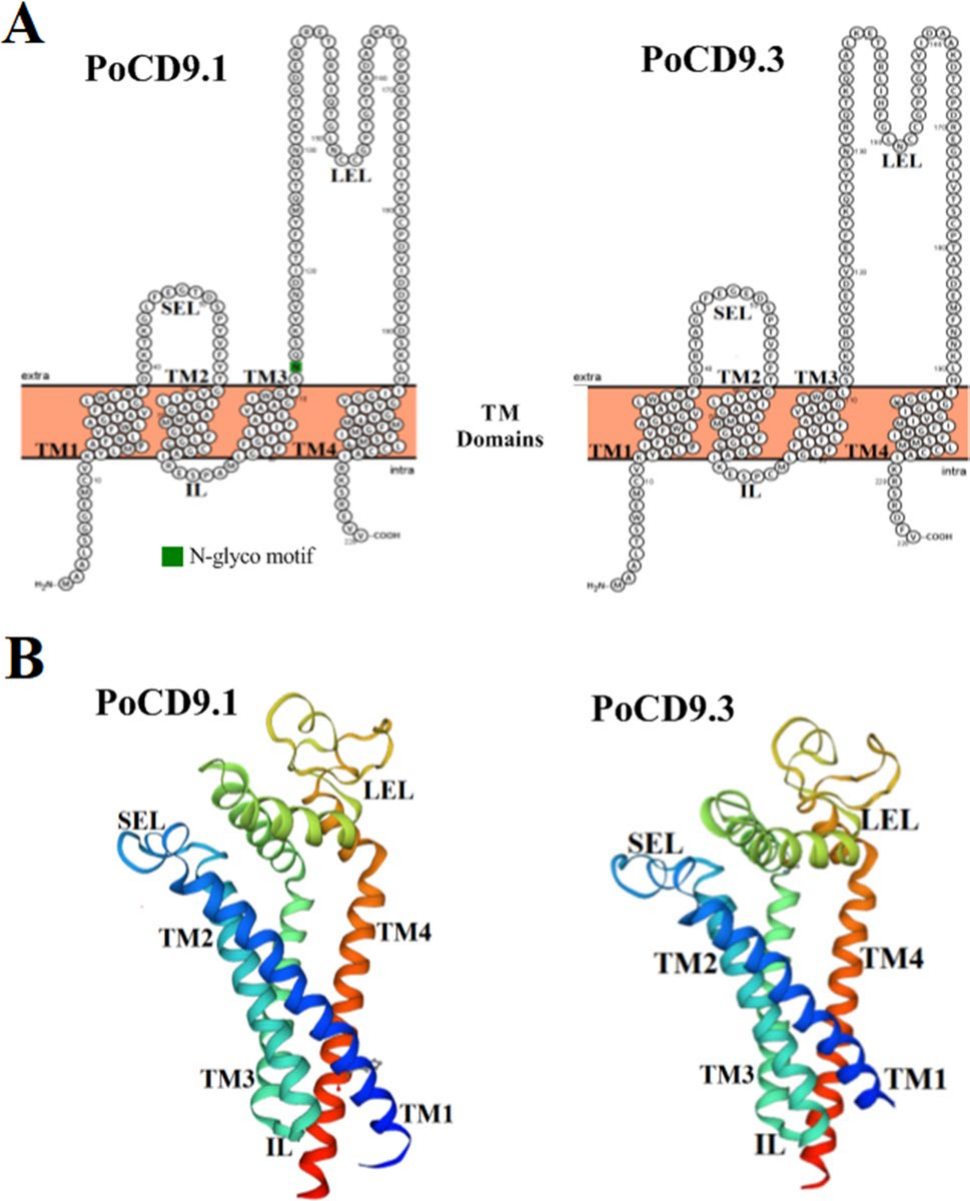
**Structures of PoCD9.1 and PoCD9.3. A**, The visualization proteoforms of PoCD9.1 and PoCD9.3 made by Protter. **B**, The three-dimensional (3D) structures of PoCD9.1 and PoCD9.3 modelled by SWISS-MODEL. The four transmembrane (TM 1–4) domains flank the small and large extracellular loops, as well as the intracellular loop (SEL, LEL, and IL, respectively).

The cDNA sequence of *PoCD9.1* contains a 687 bp open reading frame (ORF), which encodes 228 amino acid residues with a calculated molecular mass of 24.9 kDa and a theoretical pI of 5.22. *PoCD9.3* has a 678 bp ORF that codes for 225 amino acid residues with a calculated molecular mass of 24.7 kDa and a theoretical pI of 5.42. The multiple sequence alignment showed that PoCD9.1 and PoCD9.3 share high and moderate overall amino acid sequence identities with CD9 homologues of bony fish (Figure 1). The sequence identity between PoCD9.1 and PoCD9.3 is 70.61%. Both proteins have characteristic structures, including conserved CD9 motifs (CCG motifs), four putative transmembrane domains (TMs), one small extracellular loop (SEL) between TM1 and TM2, and one large extracellular loop (LEL) between TM3 and TM4 (Figure 1 and Figure 2). Protein homologous modelling was performed to generate the three-dimensional (3D) structure of PoCD9.1 and PoCD9.3 based on their amino acid sequences and human tetraspanin CD9 (PDB code: 6k4j) [46]. The 3D structures of PoCD9.1 and PoCD9.3 are also highly similar, including six α helices (Figure 2B). SEL and LEL are outside the cell, and the intracellular loop (IL) joins TM2 and TM3. The C-terminal domain and the N-terminal domain are located in the cytoplasm (Figure 2). However, PoCD9.1 has an N-glycosylation motif, but PoCD9.3 does not.

**Figure 3:**
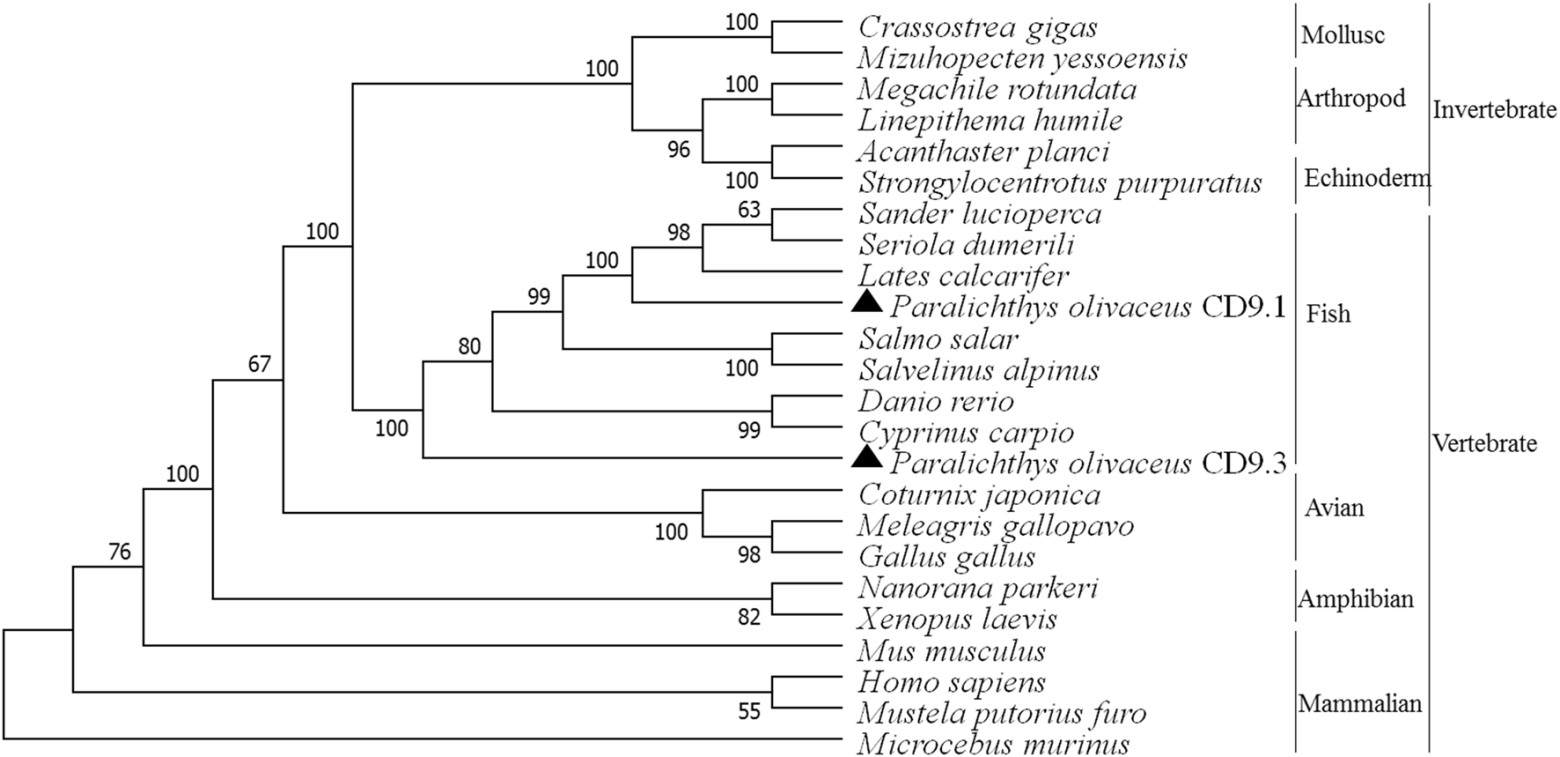
**Phylogenetic analysis of PoCD9.1 and PoCD9.3.** The phylogenetic tree was constructed with MEGA 6.0 software using the neighbour-joining method. For confidence values for the phylogenetic analysis, 1000 bootstrap trials were performed. The black triangle indicates the CD9 protein of *Paralichthys olivaceus*. The numbers at the forks indicate the bootstrap value. Species and their protein accession numbers are as follows: *Seriola dumerili* (XP_022610189.1), *Lates calcarifer* (XP_018519439.1), *Sander lucioperca* (XP_031149225.1), *P. olivaceus* CD9.1 (019,934,457.1), *Salmo salar* (XP_014003925.1), *Salvelinus alpinus* (XP_023827461.1), *Danio rerio* (NP_997784.1), *Cyprinus carpio* (KTF92709.1), *P. olivaceus* CD9.3 (XP_019964847.1), *Nanorana parkeri* (XP_018418195.1), *Xenopus laevis* (NP_001085461.1), *Meleagris gallopavo* (XP_031411372.1), *Gallus gallus* (NP_990093.1), *Coturnix japonica* (XP_015726988.1), *Homo sapiens* (NP_001760.1), *Mustela putorius furo* (XP_004766790.1), *Microcebus murinus* (XP_012616069.1), *Mus musculus* (NP_031683.1), *Crassostrea gigas* (XP_011430305.1), *Mizuhopecten yessoensis* (XP_021353101.1), *Acanthaster planci* (XP_022082820.1), *Strongylocentrotus purpuratus* (XP_030845089.1), *Megachile rotundata* (XP_003699639.1), and *Linepithema humile* (XP_012235518.1).

To investigate the evolutionary relationship of PoCD9.1 and PoCD9.3 with their counterparts, we generated a phylogenetic tree based on the amino acid sequences of CD9 homologs (Figure 3). The phylogenetic analysis was performed using the neighbour-joining (NJ) method of the MEGA 7 program, and the results showed that multiple tetraspanins were separated into two main branches: invertebrates (three subbranches, mollusks, echinoderms, and arthropods) and vertebrates (four subbranches, fish, avians, amphibians, and mammals). PoCD9.1 forms a group with CD9 homologs of *L. calcarifer*, *Seriola dumerili*, and *Sander lucioperca*. However, PoCD9.3 forms a separate group in the fish subbranch.

### Expression of PoCD9.1 and PoCD9.3 under normal physiological conditions

**Figure 4:**
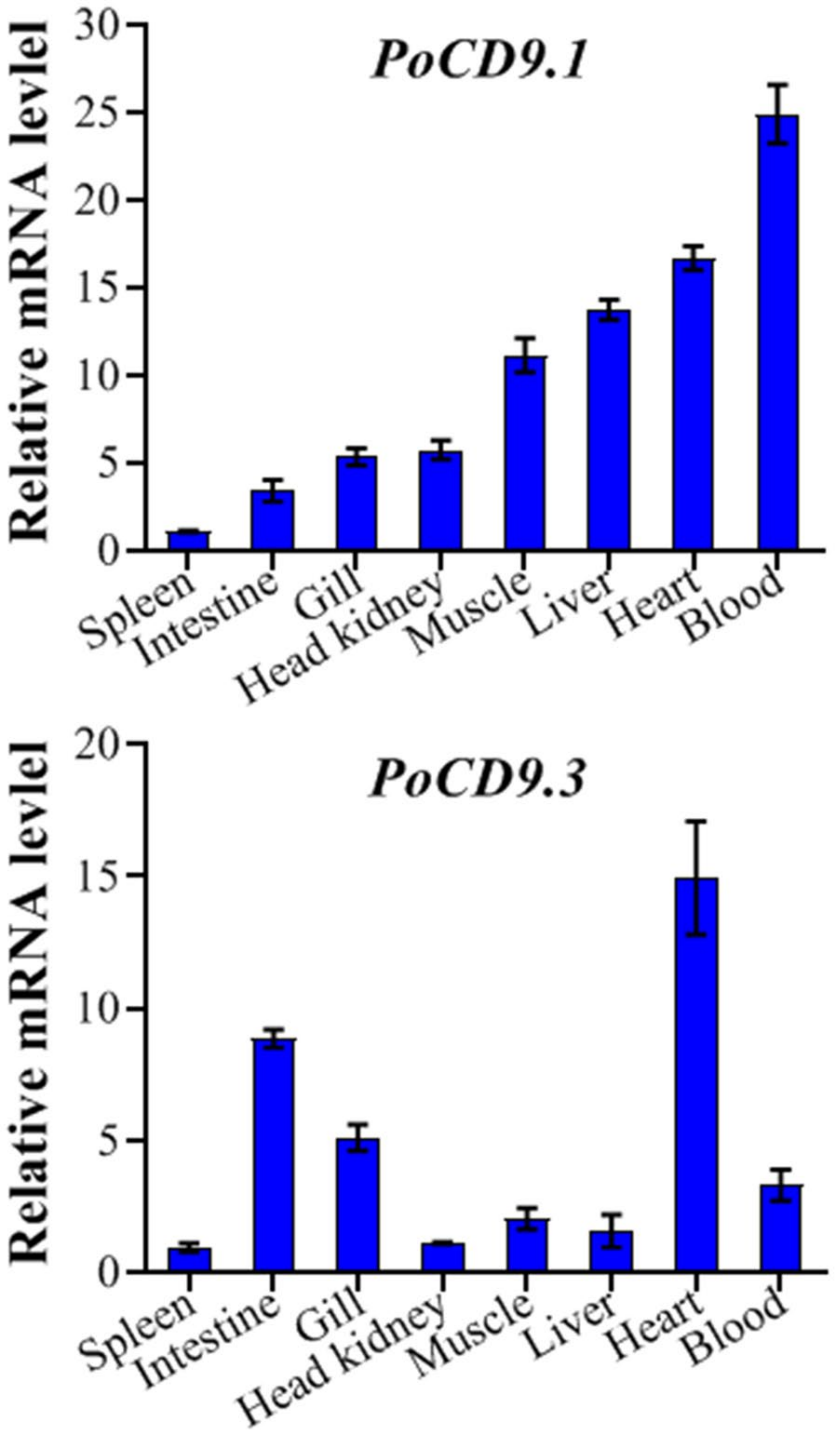
**Expression of PoCD9.1 and PoCD9.3 in flounder tissues.** *PoCD9.1* expression (**A**) and *PoCD9.3* expression (**B**) in the muscle, spleen, blood, gill, heart, head kidney, liver, and intestine of flounder were determined by quantitative real-time RT-PCR. For convenience of comparison, the expression level in the spleen was set as 1 because its expression level was the lowest. Data are the mean of three independent assays and presented as the mean ± SEM (*N* = 3). N represents the number of times the experiment was performed.

There are some differences between the amino acid sequences of PoCD9.1 and PoCD9.3, but their secondary structures and tertiary structures are highly similar, so we wanted to explore whether the two CD9 proteins exhibit functional inconsistencies. First, we examined the expression profiles of *PoCD9.1* and *PoCD9.3* under normal physiological conditions. With this consideration, RT-qPCR was carried out, and the results showed that *PoCD9.1* and *PoCD9.3* were ubiquitously expressed in all examined tissues (Figure 4). The lowest expression of both *CD9* molecules occurred in the spleen. However, the tissues with the highest expression of the two *CD9* homologs were different. For *PoCD9.1*, the highest expression was observed in the blood, followed by the heart and liver. The expression of *PoCD9.3* was highest in the heart, followed by the intestine and gill. The similarities and differences of both CD9 expression profiles indicate their functional consistency and specificity.

### Expression profiles of PoCD9.1 and PoCD9.3 upon experimental infection with bacterial and viral pathogens

**Figure 5:**
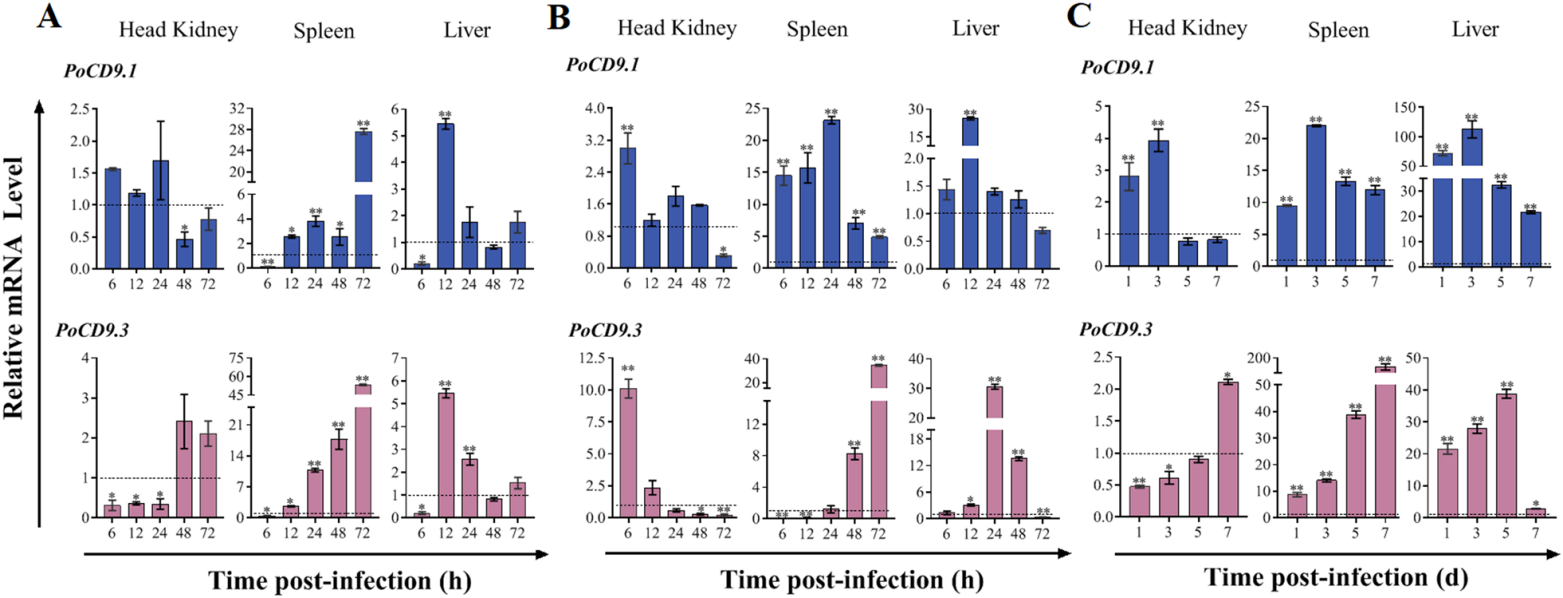
***PoCD9.1*** **and** ***PoCD9.3*** **expression in response to pathogen challenge**. *Vibrio anguillarum* and *Edwardsiella piscicida* were cultured in LB broth at 28 °C to an optical density of 0.8 at 600 nm. Then, the cells were washed with PBS and resuspended in PBS to a concentration of 5 × 10^6^ CFU. ISKNV was resuspended in PBS to a concentration of 1 × 10^6^ copies/mL. Flounders were injected intraperitoneally with 50 μL of *V. anguillarum* (**A**), *E. piscicida* (**B**), ISKNV (**C**), or PBS. After infection, the head kidney, spleen, and liver from three fish were taken aseptically at 6, 12, 24, 48, and 72 h post-infection (hpi) for bacterial infection and at 1, 3, 5, and 7 days post-infection (dpi) for viral infection. *PoCD9.1* and *PoCD9.3* expression in the three tissues was determined by RT-qPCR at various time points. In each case, the expression level at 0 h was set as 1. Values are shown as the mean ± SEM (*N* = 3). N represents the number of times the experiment was performed. *, *P* < 0.05; **, *P* < 0.01.

Next, we wanted to explore the involvement of *PoCD9.1* and *PoCD9.3* in the flounder immune response. For this purpose, fish were challenged experimentally with the extracellular pathogen *V. anguillarum*, the intracellular pathogen *E. piscicida*, and the viral pathogen ISKNV. Total RNA was extracted from three cardinal immune tissues at different time points, and cDNA was synthesized. Then, the expression levels of *PoCD9.1* and *PoCD9.3* were determined by RT-qPCR. The results showed that the expression patterns of *PoCD9.1* and *PoCD9.3* appeared to depend on the nature of the pathogen, tissue type, and infection time (Figure 5).

Specifically, upon infection with the extracellular pathogen *V. anguillarum*, as shown in Figure 5A, *PoCD9.1* expression in the head kidney was basically unchanged, and *PoCD9.3* expression in the head kidney was downregulated at 6, 12, and 24 hpi and then returned to the normal level. In the spleen, *PoCD9.1* and *PoCD9.3* expression was significantly decreased at 6 hpi and then gradually increased and peaked at 72 hpi (27.63- and 53.25-fold, respectively). In the liver, *PoCD9.1* and *PoCD9.3* expression was significantly downregulated at 6 hpi but was enhanced after 6 hpi. The peak values were observed at 12 hpi (5.45- and 5.37-fold, respectively) and then gradually returned to the normal level. From the perspective of infection with the extracellular pathogen *V. anguillarum*, the spleen is the most important tissue among the three immune tissues for the effects of *PoCD9.1* and *PoCD9.3* against infection.

When the fish were infected with the intracellular bacterial pathogen *E. piscicida*, the expression levels of *PoCD9.1* and *PoCD9.3* in the head kidney were similar, i.e., they were significantly upregulated in the early stage of infection and then fell back or were even downregulated (Figure 5B). However, in the spleen, the expression of the two *CD9* homologs was completely different. For *PoCD9.1*, its expression was markedly upregulated and peaked at 24 hpi (23.84-fold); for *PoCD9.3*, its expression was significantly downregulated before 24 hpi and then substantially increased after 24 hpi, with maximum induction detected at 72 hpi (34.8-fold). In the liver, the expression of *PoCD9.1* was upregulated only at 12 hpi (25.03-fold), while the expression of *PoCD9.3* was significantly upregulated at 12, 24, and 48 hpi, peaked at 24 hpi (31.4-fold) and was downregulated at 72 hpi. These results seemingly indicate that in the early stage of infection, *PoCD9.1* is critical for the control of *E. piscicida* invasion. However, during the middle or late stage of infection, *PoCD9.3* plays a more important role in anti-*E. piscicida* infection. From the perspective of intracellular bacterial *E. piscicida* infection, the spleen is also the most important tissue among the three immune tissues for the anti-infectious effects of *PoCD9.1* and *PoCD9.3.*

During ISKNV infection, *PoCD9.1* expression in the head kidney was significantly upregulated at 1 and 3 dpi and then returned to a normal level, with maximum induction detected at 3 dpi (3.94-fold) (Figure 5C). Unlike *PoCD9.1*, *PoCD9.3* expression in the head kidney was significantly downregulated at 1 and 3 dpi and then gradually increased, and the maximum induction was 2.11-fold at 7 dpi. In the spleen, *PoCD9.1* and *PoCD9.3* expression was significantly upregulated at all examined time points, and the maximum expression of *PoCD9.1* was 22.01-fold at 3 dpi, while that of *PoCD9.3* was 175.83-fold at 7 dpi, which suggests that in the spleen, *PoCD9.3* might play more important roles in fighting against IKSNV infection than *PoCD9.1* during the middle and late stages of infection. Similar to the results in the spleen, *PoCD9.1* and *PoCD9.3* expression in the liver was significantly enhanced at all examined time points and peaked at 3 dpi (112.87-fold) and 5 dpi (38.83-fold), respectively. For *PoCD9.1*, the liver may be the more important tissue than the spleen for its immune function in defence against IKSNV infection, and for *PoCD9.3*, the spleen may be the more important tissue.

### PoCD9.1 and PoCD9.3 knockdown and its effect on resistance against bacterial infection

#### Knockdown of *PoCD9.1 *and *PoCD9.3*

**Figure 6:**
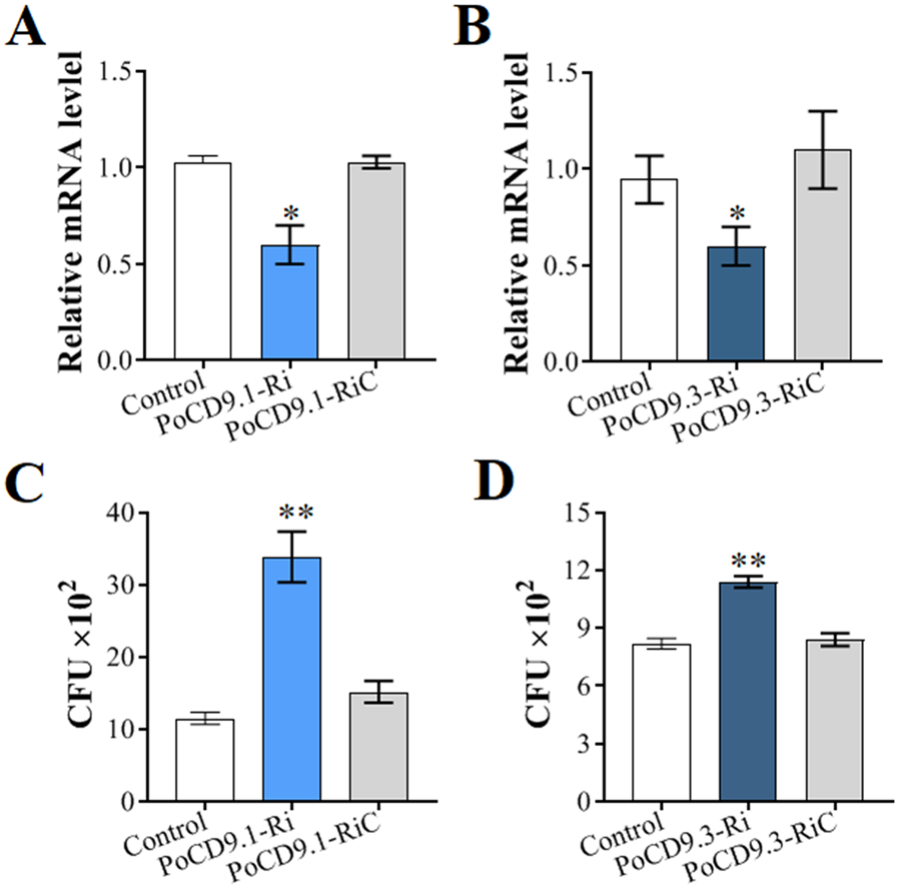
**Effect of PoCD9 knockdown on bacterial infection.** PoCD9.1-Ri, PoCD9.1-RiC, PoCD9.3-Ri, and PoCD9.3-RiC (RNAi control) were synthesized and transfected into FG cells, and the expression of *PoCD9.1* and *PoCD9.3* was determined by RT-qPCR (**A, B**). After transfection, the FGs were infected with *Edwardsiella piscicida* for 6 h, and the amounts of bacteria were determined (**C, D**). Control, the cells treated with PBS. Values are shown as the mean ± SEM (*N* = 3). N, the number of times the experiment was performed. *, *P* < 0.05; **, *P* < 0.01.

As observed above, PoCD9.1 and PoCD9.3 could participate in the immune defence response of flounder against pathogenic infection. To analyse their effects on host defence against bacterial infection, we further examined the effects of *PoCD9.1* and *PoCD9.3* knockdown on bacterial invasion. For this purpose, PoCD9.1-Ri, PoCD9.1-RiC (RNAi control), PoCD9.3-Ri, and PoCD9.3-RiC (RNAi control) were synthesized and transfected into FG cells. The expression levels of *PoCD9.1* and *PoCD9.3* were determined by RT-qPCR, and the results showed that in the PoCD9.1-Ri-treated cells, the expression of *PoCD9.1* was significantly reduced compared to that in the control cells. The expression of *PoCD9.1* in the PoCD9.1-RiC-treated cells was comparable to that in the PBS-treated cells (NC) (Figure 6A). Similar results were observed in the PoCD9.3-Ri- or PoCD9.3-RiC-administered cells (Figure 6B).

#### Effects of *PoCD9.1 and PoCD9.3* knockdown on resistance against bacterial infection

To examine the effects of *PoCD9.1* and *PoCD9.3* knockdown on host cell defence against bacterial pathogens, we incubated the FG cells treated with PoCD9.1-Ri or PoCD9.3-Ri with *E. piscicida*, and the bacterial numbers were determined at 6 hpi. The results showed that the cells administered PoCD9.1-Ri exhibited significantly increased bacterial amounts compared to the control cells, whereas the cells administered PoCD9.1-RiC exhibited bacterial amounts comparable to those in the control cells (Figure 6C). Similar results were observed in the PoCD9.3-Ri- or PoCD9.3-RiC-administered cells (Figure 6D). Moreover, compared with those of their respective controls, increased bacterial amounts in the PoCD9.1-Ri-administered cells were higher than those in the PoCD9.3-Ri-administered cells. In this context, *PoCD9.1* knockdown was more lethal than *PoCD9.3* knockdown for host cells with *E. piscicida* infection.

### The effects of PoCD9.1 and PoCD9.3 overexpression on defence against pathogens

#### Overexpression of *PoCD9.1* and *PoCD9.3*

**Figure 7:**
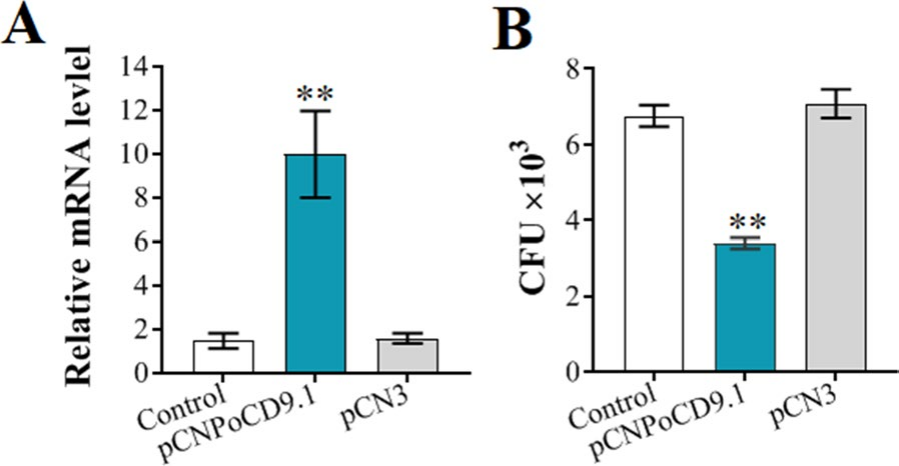
**Overexpression of PoCD9.1 and its effect on antibacterial infection in FG cells.** Overexpression of PoCD9.1. FG cells were administered pCNPoCD9.1, pCN3, and PBS. At 24 h post-transfection, RNA was extracted from the FGs and used for RT-qPCR with primers specific to *PoCD9.1*. Data are shown as the mean gene expression relative to the expression of the endogenous control β-actin (**A**). After transfection, the FGs were infected with *E. piscicida* for 6 h, and the amounts of bacteria were determined (**B**). Values are shown as the mean ± SEM (*N* = 3). N, the number of times the experiment was performed. *, *P* < 0.05, **, *P* < 0.01.

Since, as observed above, *PoCD9.1* and *PoCD9.3* knockdown attenuated the resistance of host cells to pathogenic infection, we further examined the effects of *PoCD9.1* and *PoCD9.3* overexpression on bacterial invasion. For this purpose, the eukaryotic expression plasmids pCNPoCD9.1 and pCNPoCD9.3, which constitutively expressed *PoCD9.1* and *PoCD9.3*, respectively, were constructed. The plasmid pCNPoCD9.1 was transfected into FG cells, and the expression of *PoCD9.1* was determined by RT-qPCR. The results showed that in the pCNPoCD9.1-administered cells, the expression of *PoCD9.1* was significantly upregulated compared to that in the control cells, which were the pCN3- or PBS-administered cells (Figure 7A).

**Figure 8:**
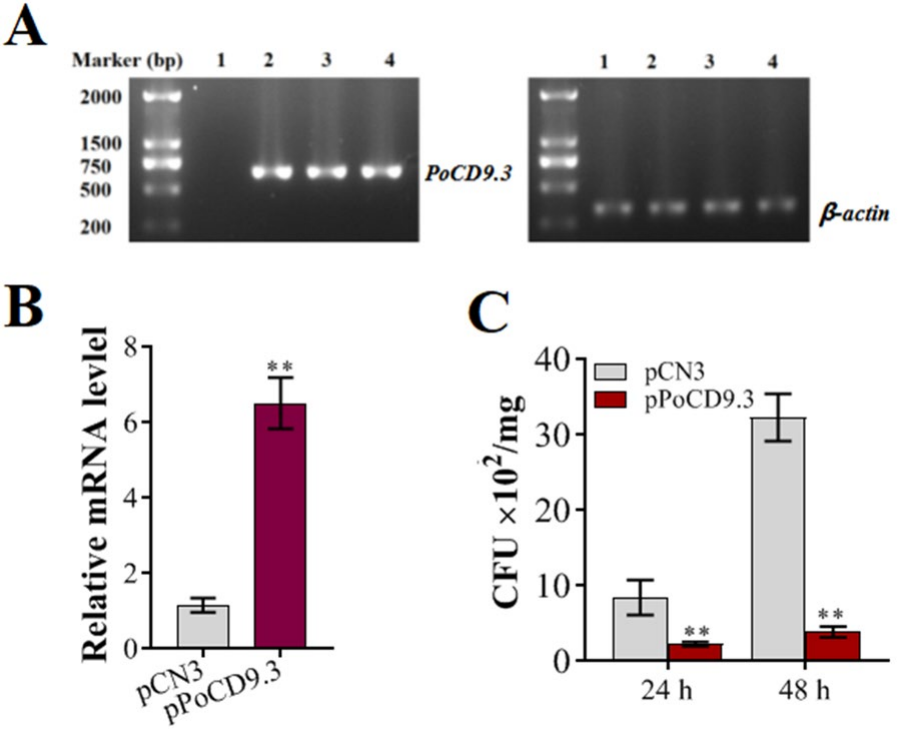
**Overexpression of PoCD9.3 and its effect on antibacterial infection in fish.** Flounders were administered pCN3 (lane 1) and pCNPoCD9.3 (lanes 2–4). Muscle tissues were taken at 5 days post-administration and used for DNA extraction, and then, PCR was performed using primers specific to pCN3, PoCD9.3, and β-actin (internal control) (**A**). RNA was extracted from the spleen and used for RT-qPCR with primers specific to PoCD9.3. Data are shown as the mean gene expression relative to the expression of the endogenous control β-actin (**B**). Flounders were administered pCN3 and pCNPoCD9.3, and at 5 days post-administration, the fish were infected with *E. piscicida*. Bacterial amounts in the spleen were determined at 24 h and 48 h post-infection (**C**). Data are presented as the mean ± SEM (*N* = 3). N, the number of times the experiment was performed. *, *P* < 0.05; **, *P* < 0.01.

For *PoCD9.3*, we wanted to observe the effect of its overexpression at the individual level. For this purpose, the fish were injected with pCNPoCD9.3 or the control plasmid pCN3, which verified that pCN3 and recombinant plasmid-based pCN3 can stably exist and be expressed in flounder and other species of fish [47–49]. At 5 days post-administration, the distribution of the plasmids was assessed by PCR, and the expression of *PoCD9.3* was examined by RT-qPCR. The PCR results showed that the plasmid pCNPoCD9.3 was detected in the muscle of the fish administered the pCNPoCD9.3 plasmids (Figure 8A and data not shown). RT-qPCR showed that the expression level of *PoCD9.3* in the spleen of the pCNPoCD9.3-administered fish was significantly higher than that of the pCN3-administered fish (Figure 8B). These results indicated that *PoCD9.3* carried on pCNPoCD9.3 was successfully expressed in fish tissues.

#### Potential of *PoCD9.1* and *PoCD9.3* overexpression against bacterial infection

To examine the effect of *PoCD9.1* overexpression on bacterial infection, we infected the FG cells treated with pCNPoCD9.1, pCN3, or PBS with *E. piscicida*, and the bacterial numbers were determined at 6 hpi. The results showed that the cells administered pCNPoCD9.1 exhibited significantly decreased bacterial amounts compared to the control cells, whereas the cells administered pCN3 exhibited bacterial amounts comparable to those in the control cells (Figure 7B).

As shown in Figure 5, the spleen was the most important tissue among the three immune tissues for *PoCD9.3* to fight against *E. piscicida* infection, especially during the middle or late stage of *E. piscicida* infection, so we examined the effect of *PoCD9.3* overexpression in the spleen on bacterial infection. For this purpose, the fish were infected with *E. piscicida* at 5 days post-plasmid administration, and the bacterial numbers in the spleen were determined at 24 and 48 hpi. The results showed that the bacterial numbers in the pCNPoCD9.3-administered fish were significantly lower than those in the pCN3-administered fish at both time points (Figure 8C).

## Discussion

With the development of research on the host immune system, an increasing number of nontraditional immune proteins have been found to participate in defence against pathogens. For example, CD9 is described as a “molecular facilitator” [50], and the biological functions of CD9 are highly dependent on the multitude of dynamic interactions that this molecule can establish with other transmembrane and cytoplasmic proteins within tetraspanin-enriched microdomains (TEMs) [51, 52]. It was reported that CD9 in TEMs plays an important role, either directly or indirectly, in the activity of numerous transmembrane and intracellular proteins, such as metalloproteinases, ion channels, receptors for growth factors, cytokines and chemokines, transporters, signalling transducers, and cytoskeletal linkers [53–58]. Here, to determine the immunological role of CD9 in teleosts, we characterized two isoforms of CD9 from Japanese flounder, PoCD9.1 and PoCD9.3, and examined their expression and biological properties.

Multiple alignment analysis revealed that PoCD9.1 and PoCD9.3 share high or moderate identity (56%–91%) with CD9 homologs of other teleost fish. The high sequence identity, together with the conserved CD9 structural features, demonstrated that PoCD9.1 and PoCD9.3 are members of the teleost TM4SF family. However, PoCD9.1 shares only 70% identity with PoCD9.3. Phylogenetic analysis showed that PoCD9.1 and PoCD9.3 have some evolutionary differences, which indicates that the two CD9 isoforms possess functional consistency and specificity.

Tissue-specific expression analysis showed that each of the two CD9 homologs was expressed in all eight tissues examined, although the expression levels were different. The lowest expression of both *CD9* molecules occurred in the spleen. However, the tissue with the highest expression of PoCD9.1 was blood, and PoCD9.3 was highly expressed in the heart, which was consistent with a previous study in which CD9 was identified as a lymphohaematopoietic marker and shown to be abundantly expressed in haematopoietic cells [18]. Similarly, the highest expression of CD9 was reported in PBLs of rainbow trout [31]. Unlike our results, in the cartilaginous fish red stingray, the strongest expression of the CD9 homologue was detected in the spleen [59]. These findings indicate that CD9 in different species possesses functional specificity.

Increasing evidence indicates that CD9 in mammals participates in the immune response to pathogenic infection [60–63]. In teleosts, similar results are also reported. For example, infection with the viral pathogen VHSV significantly upregulated the expression of CD9 in rainbow trout [32]. In flounder, CD9 is one of the upregulated genes after inflammatory stimuli or VHSV infection [64, 65]. To systematically explore the immune response of *PoCD9.1* and *PoCD9.3* upon fish pathogen infection, we challenged flounder with the extracellular pathogen *V. anguillarum*, intracellular pathogen *E. piscicida*, and viral pathogen ISKNV, and the expression of *PoCD9.1* and *PoCD9.3* in three immune tissues was examined. Our results showed, in general, that the expression of *PoCD9.1* and *PoCD9.3* was enhanced upon infection with the three pathogens. In the case of *V. anguillarum* infection, the expression of *PoCD9.1* and *PoCD9.3* in the spleen was dramatically increased, although the spleen was the tissue with the lowest levels under normal conditions. These results indicated that the spleen was perhaps the most important tissue among the three immune tissues for *PoCD9.1* and *PoCD9.3* to fight against *V. anguillarum* infection. In general, the *V. anguillarum*-induced expression of *PoCD9.1* was higher than that of *PoCD9.3* in the three tissues.

When flounders were infected with the intracellular bacterial pathogen *E. piscicida*, the spleen was also the most important tissue, in which *PoCD9.1* and *PoCD9.3* played an important role in anti-infectious immunity. However, the two CD9 homologs functioned in different periods. At the early stage of *E. piscicida* infection, *PoCD9.1* expression in the spleen was markedly upregulated, which indicated that PoCD9.1 was critical for controlling *E. piscicida* invasion; in contrast, *PoCD9.3* expression in the spleen was dramatically downregulated. One of the reasons might be the immune escape strategy implemented by the pathogen since *E. piscicida* has the capacity to inhibit host immune defence [66]. In the late stage of *E. piscicida* infection, *PoCD9.1* expression in the spleen was decreased, but *PoCD9.3* expression was substantially enhanced. During ISKNV infection, there are differences in both *PoCD9.1* and *PoCD9.3* expression. In the spleen, *PoCD9.3* might play more important roles in fighting IKSNV infection than *PoCD9.1* at the middle and late stages of infection. These results suggest that PoCD9.1 and PoCD9.3 display different anti-infectious functions at different stages of pathogenic infection.

A recent report showed that cellular depletion of CD9 tetraspanins could reduce HPV16 infection in HeLa cells, and CD9 was identified as a key cellular factor for HPV16 infection [67]. The knockdown of CD9 significantly reduced the adherence of *Neisseria meningitidis* to host cells [68]. Peptides from the tetraspanin CD9 are potent inhibitors of *Staphylococcus aureus* adherence to keratinocytes [69]. Currently, there is no functional report on CD9 in immune defence against pathogenic infection in teleosts. Similar to the results in mammalian species, in flounder, knockdown of *PoCD9.1* and *PoCD9.3* significantly weakened the capacity of host cells to clear *E. piscicida*. Moreover, *PoCD9.1* knockdown was more lethal than *PoCD9.3* knockdown in host cells against *E. piscicida* infection. To further clarify the immunological functions, we overexpressed *PoCD9.1*, and the FG cells with *PoCD9.1* overexpression displayed fewer bacteria than the control cells with *E. piscicida* infection. Consistent with the in vitro results, the in vivo findings showed that the *PoCD9.3*-overexpressing flounder exhibited significantly lower bacterial amounts than the control fish. These results, together with those of the expression analysis, indicate a positive role of *PoCD9.1* and *PoCD9.3* in host immunity against pathogenic infection.

In conclusion, we reported for the first time the immunological function of the teleost CD9 homologs PoCD9.1 and PoCD9.3 from Japanese flounder. The expression of *PoCD9.1* and *PoCD9.3* was significantly induced by extracellular and intracellular bacterial pathogens and viral pathogens, which indicates that the two CD9 homologs play an important role in the response to pathogenic infection. Among the three cardinal immune tissues, the spleen was the main tissue of PoCD9.1 and PoCD9.3 in response to extracellular and intracellular pathogen infection. PoCD9.1 and PoCD9.3 displayed different anti-infectious functions at different stages of pathogenic infection. The knockdown of *PoCD9.1* and *PoCD9.3* attenuated the ability of host cells to eliminate pathogenic bacteria, which was confirmed by the finding that overexpression of *PoCD9.1* or *PoCD9.3* promoted host cells or host defence against invading pathogenic microorganisms. These findings add new insights to the biological function of teleost CD9.

## Supplementary Information

**Additional file 1.** **Expression stability of beta-actin.** Vibrio anguillarum and Edwardsiella piscicida were cultured in LB broth at 28 °C to an optical density of 0.8 at 600 nm. Then, the cells were washed with PBS and resuspended in PBS to a concentration of 5 × 106 CFU. ISKNV was resuspended in PBS to a concentration of 1 × 106 copies/mL. Flounders were injected intraperitoneally with 50 μL of V. anguillarum, E. piscicida, ISKNV, or PBS. After infection, the head kidney, spleen, and liver from three fish were taken aseptically at 6, 12, 24, 48, and 72 hpi for bacterial infection and at 1, 3, 5, and 7 dpi for viral infection. The beta-actin expression in the three tissues was determined by RT-qPCR at various time points with EF1 alpha as the reference. In each case, the expression level at 0 h was set as 1. Values are shown as the mean ± SEM (*N* = 3). N represents the number of times the experiment was performed. *, *P* < 0.05; **, *P* < 0.01.

## Data Availability

All data generated or analysed during this study are included in this published article.

## Abbreviations

*E. piscicida*: *Edwardsiella piscicida*
*V. anguillarum*: *Vibrio anguillarum*
*E. coli*: *Escherichia coli*
ISKNV: Infectious spleen and kidney necrosis virus
*P. olivaceus*: *Paralichthys oliv*aceus
LB: Luria–Bertani broth
MEM: Minimal essential medium
BCS: Bovine calf serum
PBS: Phosphate-buffered saline
PCR: Polymerase chain reaction
RT-qPCR: Quantitative real-time reverse transcription-PCR
hpi: Hour post-infection
dpi: Day post-infection
ORF: Open reading frame
TM: Transmembrane domain
SEL: Small extracellular loop
LEL: Large extracellular loop
IL: Intracellular loop
TEMs: Tetraspanin-enriched microdomains
NJ: Neighbour-joining

## References

[1] Guan X, Zhang BC, Sun L (2019). Japanese flounder pol-miR-3p-2 suppresses *Edwardsiella tarda* infection by regulation of autophagy via p53. Dev Comp Immunol.

[2] Abayneh T, Colquhoun D, Sørum H (2012). *Edwardsiella piscicida* sp. nov., a novel species pathogenic to fish. J Appl Microbiol.

[3] Liu Y, Zhao L, Yang M, Yin K, Zhou X, Leung K, Liu Q, Zhang Y, Wang Q (2017). Transcriptomic dissection of the horizontally acquired response regulator EsrB reveals its global regulatory roles in the physiological adaptation and activation of T3SS and the cognate effector repertoire in *Edwardsiella piscicida* during infection toward turbot. Virulence.

[4] Mohanty B, Sahoo PK (2008). Edwardsiellosis in fish: a brief review. J Biosci.

[5] Bujan N, Toranzo A, Magarinos B (2018). *Edwardsiella piscicida*: a significant bacterial pathogen of cultured fish. Dis Aquat Organ.

[6] Ucko M, Colorni A, Dubytska L, Thune R (2016). *Edwardsiella piscicida*-like pathogen in cultured grouper. Dis Aquat Organ.

[7] Chen S, Ma X, Wu D, Yang D, Zhang Y, Liu Q (2019). Scophthalmus maximus interleukin-1β limits *Edwardsiella piscicida* colonization in vivo. Fish Shellfish Immunol.

[8] Yang D, Liu X, Xu W, Gu Z, Yang C, Zhang L, Tan J, Zheng X, Wang Z, Quan S, Zhang Y, Liu Q (2019). The *Edwardsiella piscicida* thioredoxin-like protein inhibits ASK1-MAPKs signaling cascades to promote pathogenesis during infection. PLoS Pathog.

[9] Stipp CS, Kolesnikova TV, Hemler ME (2003). Functional domains in tetraspanin proteins. Trends Biochem Sci.

[10] Boucheix C, Benoit P, Frachet P, Billard M, Worthington RE, Gagnon J, Uzan G (1991). Molecular cloning of the CD9 antigen. A new family of cell surface proteins. J Biol Chem.

[11] Boucheix C, Rubinstein E (2001). Tetraspanins. Cell Mol Life Sci.

[12] Levy S, Shoham T (2005). The tetraspanin web modulates immune-signalling complexes. Nat Rev Immunol.

[13] Hemler ME (2001). Specific tetraspanin functions. J Cell Biol.

[14] Hemler ME (2014). Tetraspanin proteins promote multiple cancer stages. Nat Rev Cancer.

[15] Ovalle S, Gutierrez-Lopez MD, Olmo N, Turnay J, Lizarbe MA, Majano P, Molina-Jimenez F, Lopez-Cabrera M, Yanez-Mo M, Sanchez-Madrid F, Cabanas C (2007). The tetraspanin CD9 inhibits the proliferation and tumorigenicity of human colon carcinoma cells. Int J Cancer.

[16] Wright MD, Moseley GW, van Spriel AB (2004). Tetraspanin microdomains in immune cell signalling and malignant disease. Tissue Antigens.

[17] Hemler ME (2008). Targeting of tetraspanin proteins–potential benefits and strategies. Nat Rev Drug Discov.

[18] Boucheix C, Benoit P (1988). CD9 antigen: will platelet physiology help to explain the function of a surface molecule during hemopoietic differentiation?. Nouv Rev Fr Hematol.

[19] Shaw AR, Domanska A, Mak A, Gilchrist A, Dobler K, Visser L, Poppema S, Fliegel L, Letarte M, Willett BJ (1995). Ectopic expression of human and feline CD9 in a human B cell line confers beta 1 integrin-dependent motility on fibronectin and laminin substrates and enhanced tyrosine phosphorylation. J Biol Chem.

[20] Tai XG, Toyooka K, Yashiro Y, Abe R, Park CS, Hamaoka T, Kobayashi M, Neben S, Fujiwara H (1997). CD9-mediated costimulation of TCR-triggered naive T cells leads to activation followed by apoptosis. J Immunol.

[21] Kaji K, Takeshita S, Miyake K, Takai T, Kudo A (2001). Functional association of CD9 with the Fc gamma receptors in macrophages. J Immunol.

[22] Rocha-Perugini V, Martinez del Hoyo G, Gonzalez-Granado J, Ramírez-Huesca M, Zorita V, Rubinstein E, Boucheix C, Sánchez-Madrid F (2017). CD9 regulates MHC-II trafficking in monocyte-derived dendritic cells. Mol Cell Biol.

[23] Bailey RL, Herbert JM, Khan K, Heath VL, Bicknell R, Tomlinson MG (2011). The emerging role of tetraspanin microdomains on endothelial cells. Biochem Soc Trans.

[24] Toyo-oka K, Yashiro-Ohtani Y, Park CS, Tai XG, Miyake K, Hamaoka T, Fujiwara H (1999). Association of a tetraspanin CD9 with CD5 on the T cell surface: role of particular transmembrane domains in the association. Int Immunol.

[25] Toyooka K, Tai G, Yashiro-Ohtani Y, Ahn H, Abe R, Hamaoka T, Kobayashi M, Neben S, Fujiwara H (1997). Synergy between CD28 and CD9 costimulation for naive T-cell activation. Immunol Lett.

[26] Reyes R, Monjas A, Yanez-Mo M, Cardenes B, Morlino G, Gilsanz A, Machado-Pineda Y, Lafuente E, Monk P, Sanchez-Madrid F, Cabanas C (2015). Different states of integrin LFA-1 aggregation are controlled through its association with tetraspanin CD9. Biochim Biophys Acta.

[27] Rocha-Perugini V, Gonzalez-Granado JM, Tejera E, Lopez-Martin S, Yanez-Mo M, Sanchez-Madrid F (2014). Tetraspanins CD9 and CD151 at the immune synapse support T-cell integrin signaling. Eur J Immunol.

[28] Sala-Valdes M, Ursa A, Charrin S, Rubinstein E, Hemler ME, Sanchez-Madrid F, Yanez-Mo M (2006). EWI-2 and EWI-F link the tetraspanin web to the actin cytoskeleton through their direct association with ezrin-radixin-moesin proteins. J Biol Chem.

[29] Gilsanz A, Sanchez-Martin L, Gutierrez-Lopez MD, Ovalle S, Machado-Pineda Y, Reyes R, Swart GW, Figdor CG, Lafuente EM, Cabanas C (2013). ALCAM/CD166 adhesive function is regulated by the tetraspanin CD9. Cell Mol Life Sci.

[30] Gutiérrez-López MD, Gilsanz A, Yáñez-Mó M, Ovalle S, Lafuente EM, Domínguez C, Monk PN, González-Alvaro I, Sánchez-Madrid F, Cabañas C (2011). The sheddase activity of ADAM17/TACE is regulated by the tetraspanin CD9. Cell Mol Life Sci.

[31] Fujiki K, Gauley J, Bols N, Dixon B (2002). Cloning and characterization of cDNA clones encoding CD9 from Atlantic salmon (*Salmo salar*) and rainbow trout (*Oncorhynchus mykiss*). Immunogenetics.

[32] Castro R, Abós B, González L, Aquilino C, Pignatelli J, Tafalla C (2015). Molecular characterization of CD9 and CD63, two tetraspanin family members expressed in trout B lymphocytes. Dev Comp Immunol.

[33] Zhang M, Sun L (2011). The tissue factor pathway inhibitor 1 of *Sciaenops ocellatus* possesses antimicrobial activity and is involved in the immune response against bacterial infection. Dev Comp Immunol.

[34] Zhang J, Sun L (2017). Transcriptome analysis reveals temperature-regulated antiviral response in turbot *Scophthalmus maximus*. Fish Shellfish Immunol.

[35] Sun Y, Liu CS, Sun L (2011). A multivalent killed whole-cell vaccine induces effective protection against *Edwardsiella tarda* and *Vibrio anguillarum*. Fish Shellfish Immunol.

[36] Du X, Wang GH, Yue B, Wang JJ, Gu QQ, Zhou S, Zhang M, Hu YH (2019). A novel C1q domain containing protein in black rockfish (*Sebastes schlegelii*) serves as a pattern recognition receptor with immunoregulatory properties and possesses binding activity to heat-aggregated IgG. Fish Shellfish Immunol.

[37] Tong S-L, Li H, Miao H-Z (1997). The establishment and partial characterization of a continuous fish cell line FG-9307 from the gill of flounder *Paralichthys olivaceus*. Aquaculture.

[38] Zhang J, Li YX, Hu YH (2015). Molecular characterization and expression analysis of eleven interferon regulatory factors in half-smooth tongue sole, *Cynoglossus semilaevis*. Fish Shellfish Immunol.

[39] Zhang J, Hu YH, Sun BG, Xiao ZZ, Sun L (2013). Selection of normalization factors for quantitative real time RT-PCR studies in Japanese flounder (*Paralichthys olivaceus*) and turbot (*Scophthalmus maximus*) under conditions of viral infection. Vet Immunol Immunopathol.

[40] Zheng W-J, Sun L (2011). Evaluation of housekeeping genes as references for quantitative real time RT-PCR analysis of gene expression in Japanese flounder (*Paralichthys olivaceus*). Fish Shellfish Immunol.

[41] Chi H, Bøgwald J, Dalmo RA, Zhang W, Hu YH (2016). Th17 master transcription factors RORα and RORγ regulate the expression of IL-17C, IL-17D and IL-17F in *Cynoglossus semilaevis*. Dev Comp Immunol.

[42] Du HH, Huang HQ, Si KW, Dai HF, Hu YH (2019). Granulocyte colony stimulating factor (GCSF) of Japanese flounder (*Paralichthys olivaceus*): immunoregulatory property and anti-infectious function. Fish Shellfish Immunol.

[43] Zhang BC, Zhou ZJ, Sun L (2016). pol-miR-731, a teleost miRNA upregulated by megalocytivirus, negatively regulates virus-induced type I interferon response, apoptosis, and cell cycle arrest. Sci Rep.

[44] Jiao XD, Zhang M, Hu YH, Sun L (2009). Construction and evaluation of DNA vaccines encoding Edwardsiella tarda antigens. Vaccine.

[45] Long H, Chen C, Zhang J, Sun L (2014). Antibacterial and antiviral properties of tongue sole (*Cynoglossus semilaevis*) high mobility group B2 protein are largely independent on the acidic C-terminal domain. Fish Shellfish Immunol.

[46] Umeda R, Satouh Y, Takemoto M, Nakada-Nakura Y, Liu K, Yokoyama T, Shirouzu M, Iwata S, Nomura N, Sato K, Ikawa M, Nishizawa T, Nureki O (2020). Structural insights into tetraspanin CD9 function. Nature Communications.

[47] Jiao XD, Dang W, Hu YH, Sun L (2009). Identification and immunoprotective analysis of an in vivo-induced *Edwardsiella tarda* antigen. Fish Shellfish Immunol.

[48] Wang JJ, Sun L (2015). *Edwardsiella tarda*-regulated proteins in Japanese flounder (*Paralichthys olivaceus*): identification and evaluation of antibacterial potentials. J Proteomics.

[49] Hu YH, Deng T, Sun L (2011). The Rab1 GTPase of *Sciaenops ocellatus* modulates intracellular bacterial infection. Fish Shellfish Immunol.

[50] Maecker HT, Todd SC, Levy S (1997). The tetraspanin superfamily: molecular facilitators. FASEB J.

[51] Charrin S, le Naour F, Silvie O, Milhiet PE, Boucheix C, Rubinstein E (2009). Lateral organization of membrane proteins: tetraspanins spin their web. Biochem J.

[52] Yanez-Mo M, Barreiro O, Gordon-Alonso M, Sala-Valdes M, Sanchez-Madrid F (2009). Tetraspanin-enriched microdomains: a functional unit in cell plasma membranes. Trends Cell Biol.

[53] Lozahic S, Christiansen D, Manie S, Gerlier D, Billard M, Boucheix C, Rubinstein E (2000). CD46 (membrane cofactor protein) associates with multiple beta1 integrins and tetraspans. Eur J Immunol.

[54] Rubinstein E, Le Naour F, Lagaudriere-Gesbert C, Billard M, Conjeaud H, Boucheix C (1996). CD9, CD63, CD81, and CD82 are components of a surface tetraspan network connected to HLA-DR and VLA integrins. Eur J Immunol.

[55] Te Riet J, Helenius J, Strohmeyer N, Cambi A, Figdor CG, Muller DJ (2014). Dynamic coupling of ALCAM to the actin cortex strengthens cell adhesion to CD6. J Cell Sci.

[56] Anzai N, Lee Y, Youn BS, Fukuda S, Kim YJ, Mantel C, Akashi M, Broxmeyer HE (2002). C-kit associated with the transmembrane 4 superfamily proteins constitutes a functionally distinct subunit in human hematopoietic progenitors. Blood.

[57] Shi W, Fan H, Shum L, Derynck R (2000). The tetraspanin CD9 associates with transmembrane TGF-alpha and regulates TGF-alpha-induced EGF receptor activation and cell proliferation. J Cell Biol.

[58] Yanez-Mo M, Gutierrez-Lopez MD, Cabanas C (2011). Functional interplay between tetraspanins and proteases. Cell Mol Life Sci.

[59] Zhu J, Yan K, Lu L, Peng C, Xu AJMI (2006). Molecular cloning and characterization of CD9 cDNA from cartilaginous fish, red stingray. Dasyatis akajei.

[60] Elgawidi A, Mohsin MI, Ali F, Watts A, Monk PN, Thomas MS, Partridge LJ (2020). A role for tetraspanin proteins in regulating fusion induced by *Burkholderia thailandensis*. Med Microbiol Immunol.

[61] Hu W, Song X, Yu H, Sun J, Zhao Y (2020). Released exosomes contribute to the immune modulation of cord blood-derived stem cells. Front Immunol.

[62] Lazareth H, Henique C, Lenoir O, Puelles VG, Flamant M, Bollée G, Fligny C, Camus M, Guyonnet L, Millien C, Gaillard F, Chipont A, Robin B, Fabrega S, Dhaun N, Camerer E, Kretz O, Grahammer F, Braun F, Huber TB, Nochy D, Mandet C, Bruneval P, Mesnard L, Thervet E, Karras A, Le Naour F, Rubinstein E, Boucheix C, Alexandrou A, Moeller MJ, Bouzigues C, Tharaux PL (2019). The tetraspanin CD9 controls migration and proliferation of parietal epithelial cells and glomerular disease progression. Nat Commun.

[63] Ebersole JL, Peyyala R, Gonzalez OA (2019). Biofilm-induced profiles of immune response gene expression by oral epithelial cells. Mol Oral Microbiol.

[64] Arma NR, Hirono I, Aoki T (2004). Characterization of expressed genes in kidney cells of Japanese flounder *Paralichthys olivaceus* following treatment with ConA/PMA and LPS. Fish Pathol.

[65] Hwang JY, Kwon MG, Seo JS, Do JW, Park MA, Jung SH, Ahn SJ (2016). Differentially expressed genes after viral haemorrhagic septicaemia virus infection in olive flounder (*Paralichthys olivaceus*). Vet Microbiol.

[66] Allingham M, van Buul J, Burridge K (2007). ICAM-1-Mediated, Src- and Pyk2-dependent vascular endothelial cadherin tyrosine phosphorylation is required for leukocyte transendothelial migration. J Immunol.

[67] Fast LA, Mikuličić S, Fritzen A, Schwickert J, Boukhallouk F, Hochdorfer D, Sinzger C, Suarez H, Monk PN, Yáñez-Mó M, Lieber D, Florin L (2018). Inhibition of tetraspanin functions impairs human *Papillomavirus* and *Cytomegalovirus* infections. Int J Mol Sci.

[68] Green L, Monk P, Partridge L, Morris P, Gorringe A, Read R (2011). Cooperative role for tetraspanins in adhesin-mediated attachment of bacterial species to human epithelial cells. Infect Immun.

[69] Ventress J, Partridge L, Read R, Cozens D, Macneil S, Monk P (2016). Peptides from tetraspanin CD9 are potent inhibitors of *Staphylococcus Aureus* adherence to keratinocytes. PLoS ONE.

