# Supplementary figures and images for: Two CD9 tetraspanin family members of Japanese flounder (Paralichthys olivaceus): characterization and comparative analysis of the anti-infectious immune function
Source: Vet Res. 2021 Feb 17;52:28. doi: 10.1186/s13567-021-00903-3 (PMC7890607; doi:10.1186/s13567-021-00903-3)

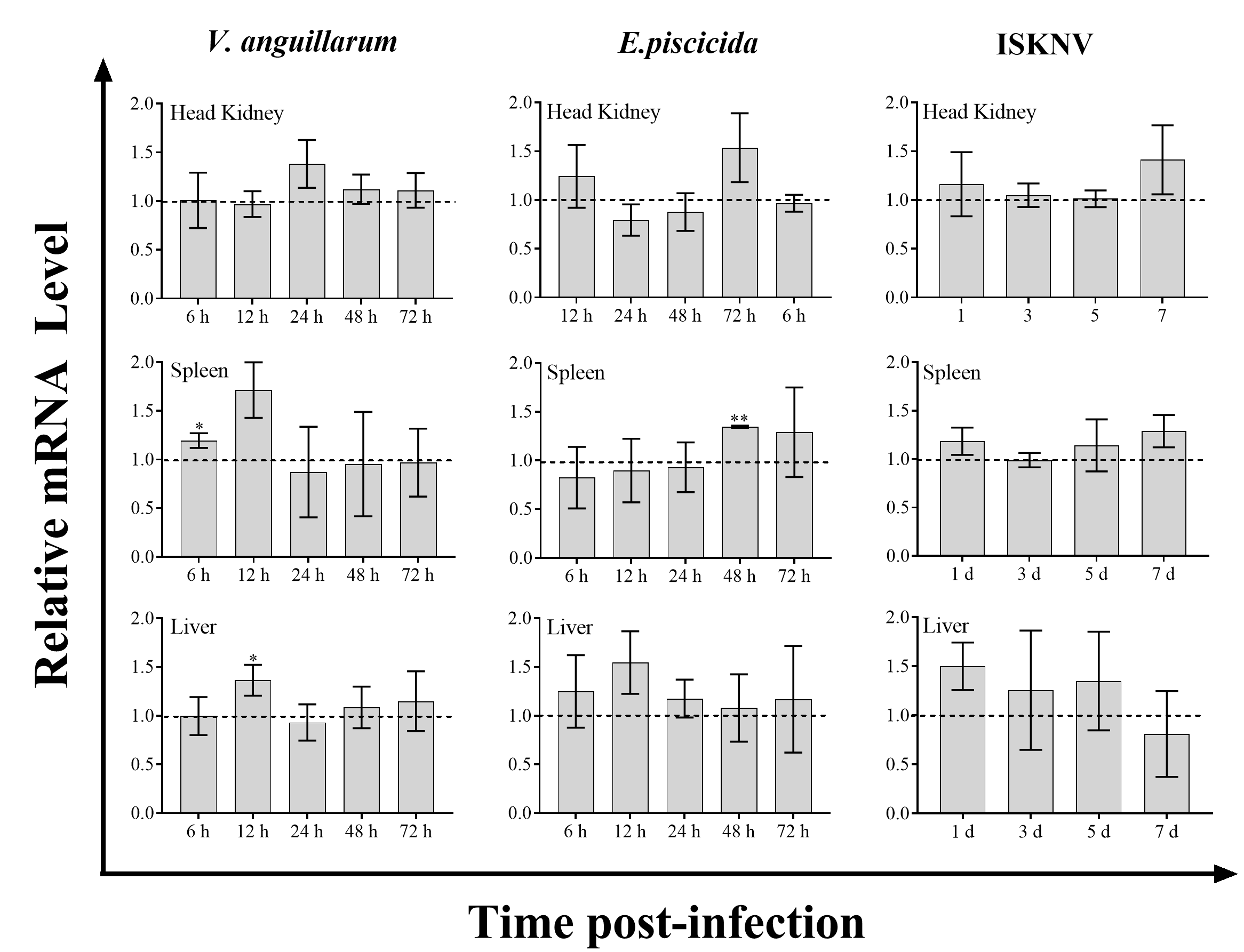

Supplement: Supplementary file 1 — Additional file 1. Expression stability of beta-actin. Vibrio anguillarum and Edwardsiella piscicida were cultured in LB broth at 28 °C to an optical density of 0.8 at 600 nm. Then, the cells were washed with PBS and resuspended in PBS to a concentration of 5 × 10^6^ CFU. ISKNV was resuspended in PBS to a concentration of 1 × 10^6^ copies/mL. Flounders were injected intraperitoneally with 50 μL of V. anguillarum, E. piscicida, ISKNV, or PBS. After infection, the head kidney, spleen, and liver from three fish were taken aseptically at 6, 12, 24, 48, and 72 hpi for bacterial infection and at 1, 3, 5, and 7 dpi for viral infection. The beta-actin expression in the three tissues was determined by RT-qPCR at various time points with EF1 alpha as the reference. In each case, the expression level at 0 h was set as 1. Values are shown as the mean ± SEM (N = 3). N represents the number of times the experiment was performed. *, P < 0.05; **, P < 0.01. [file 13567_2021_903_MOESM1_ESM.tif]
